# Who gets a family physician through centralized waiting lists?

**DOI:** 10.1186/s12875-014-0220-7

**Published:** 2015-02-05

**Authors:** Mylaine Breton, Astrid Brousselle, Antoine Boivin, Danièle Roberge, Raynald Pineault, Djamal Berbiche

**Affiliations:** Charles-LeMoyne Hospital Research Centre, Longueuil Campus, 150 Place Charles-LeMoyne, Room 200, Longueuil, J4L 0A8, Quebec Canada; Université de Sherbrooke, Sherbrooke, Quebec Canada; University of Montreal, Montreal, Quebec Canada; University of Montreal Hospital Research Centre, Montreal, Quebec Canada

**Keywords:** Payment, Incentives, Centralized waiting lists, Registry, Enrolment, Vulnerable patients

## Abstract

**Background:**

North American patients are experiencing difficulties in securing affiliations with family physicians. Centralized waiting lists are increasingly being used in Organisation for Economic Co-operation and Development countries to improve access. In 2011, the Canadian province of Quebec introduced new financial incentives for family physicians’ enrolment of orphan patients through centralized waiting lists, the *Guichet d’accès aux clientèles orphelines*, with higher payments for vulnerable patients. This study analyzed whether any significant changes were observed in the numbers of patient enrolments with family physicians’ after the introduction of the new financial incentives. Prior to then, financial incentives had been offered for enrolment of vulnerable patients only and there were no incentives for enrolling non-vulnerable patients. After 2011, financial incentives were also offered for enrolment of non-vulnerable patients, while those for enrolment of vulnerable patients were doubled.

**Methods:**

A longitudinal quantitative analysis spanning a five-year period (2008–2013) was performed using administrative databases covering all patients enrolled with family physicians through centralized waiting lists in the province of Quebec (n = 494,697 patients). Mixed regression models for repeated-measures were used.

**Results:**

The number of patients enrolled with a family physician through centralized waiting lists more than quadrupled after the changes in financial incentives. Most of this increase involved non-vulnerable patients. After the changes, 70% of patients enrolled with a family physician through centralized waiting lists were non-vulnerable patients, most of whom had been referred to the centralized waiting lists by the physician who enrolled them, without first being registered in those lists or having to wait because of their priority level.

**Conclusion:**

Centralized waiting lists linked to financial incentives increased the number of family physicians’ patient enrolments. However, although vulnerable patients were supposed to be given precedence, physicians favoured enrolment of healthier patients over those with greater health needs and higher assessed priority. These results suggest that introducing financial incentives without appropriate regulations may lead to opportunistic use of the incentive system with unintended policy consequences.

## Background

Effective health systems rely on well-organized and accessible primary healthcare [1,2]. The Pan American Health Organization has suggested renewing primary healthcare in the Americas based on a core set of functional and structural elements that guarantee access to services [3]. Over the past decade, several industrial countries, including Canada, have recommended reinforcing primary care services to guarantee every citizen access to a family physician [4-9]. Despite those recommendations, North American patients are experiencing greater difficulties in securing affiliation with a regular family physician compared to those in other OECD countries [10].

To address these difficulties in accessing continuous primary care services, particularly for more vulnerable clienteles, four Canadian provinces have created centralized waiting lists to improve access to family physicians: *Health Care Connect* in Ontario, *Healthcare NB* in New Brunswick, *A GP for Me* in British Columbia, and the *Guichet d’accès aux clientèles orphelines* (GACO) in Quebec. These waiting lists centralize requests for family physicians in a given territory and match unattached patients with family physicians based on a priority scale and on the availability of primary care resources [11].

In these four provinces, patient enrolment through centralized waiting lists is associated with financial incentives for family physicians [12]. Several other health policies also use financial incentives to encourage physician buy-in [13-15]. One of the best-known models is pay-for-performance, which uses financial incentives to reward professionals who meet quality standards [16]. The greatest challenge in using financial incentives is to carefully define and effectively align the modalities with the targeted objectives to avoid unintended consequences, such as over- or under-utilization of services [17].

### Objective

The objective of this study was to analyze whether there were any significant changes in patient enrolments with family physicians through GACOs in Quebec after the introduction of new financial incentives. The numbers of patients were analyzed according to various patient characteristics, such as vulnerability criteria, assessed priority level, and source of registration in the GACO. We analyzed data at the provincial level over a five-year period (2008–2013), before and after the introduction of new financial incentives in November 2011. The centralized waiting lists (GACOs) implemented in 2008 for patients without family physicians had two objectives: 1) to increase the number of persons with a family physician, and 2) to give priority to vulnerable patients. Financial incentives were provided from the outset in 2008, which were then modified in November 2011. The results of our study allowed us to analyze the changes in family physicians’ enrolment of orphan or unattached patients (i.e., those without a family physician) and to determine whether vulnerable patients were given prioritized access to a family physician.

## Methods

### Setting

Quebec is a province of over eight million residents with a tax-based system providing universal insurance coverage for medical services. In this system, physicians are remunerated predominantly on a fee-for-service basis, with those fees also covering operating costs. Private practices are operated by family physicians, who are self-employed. There are also government-operated medical clinics, called local community health centres, where family physicians are paid on a salary basis, but less than 20% of family physicians work in such centres. New models of primary care, such as Family Medicine Groups (FMGs) and Network Clinics, were put in place in the early 2000s. These new models, intended to improve healthcare organization and delivery, are based on contractual agreements with the government [18]. FMGs are groups of physicians working in close collaboration with nurses to provide services to registered patients. The government pays for the recruitment of nurses and administrative staff and the acquisition of computer equipment [19]. As of January 2014, there were over 250 accredited FMGs in Quebec, in which more than 40% of the province’s residents were enrolled.

Formal enrolment of patients with physicians is relatively new, and patient enrolment payments represent only a marginal proportion of physicians’ income in Quebec. Enrolment payments were instituted in the early 2000s with the introduction of FMGs. At that time, only family physicians practising in FMGs received an annual bonus for enrolling non-vulnerable patients. It was only recently, in 2009, that payments for enrolment of non-vulnerable patients were extended to all other family physicians (i.e., those not in FMGs). These payments are modulated based on clientele characteristics and the model of primary care. They constitute the beginnings of a capitation model, but without penalties or any requirements for physicians to see their patients, or for their patients to consult them, in order to receive payment. These annual payments are approximately $10 per non-vulnerable patient and $55 per vulnerable patient, plus a per-visit surcharge for the vulnerable patients [20].

### The policy intervention: centralized waiting lists for orphan patients

Quebec has the highest rate (28%) in Canada of persons reporting not having a family physician [21]. In response to this substantial need to connect orphan patients with family physicians, the Ministry of Health and Social Services (MSSS) and the Quebec Federation of General Practitioners (FMOQ) jointly decided, in 2008, to implement 93 centralized waiting lists for unattached patients, the *Guichets d’accès aux clientèles orphelines* (GACO). These GACOs are managed by the Health and Social Services Centres, which are responsible for the population in their territory. The objective of these GACOs is to facilitate access for the territory’s residents to a family physician, based on both a clinical priority scale and medical manpower availability. Each GACO is run by a nurse and an administrative assistant, in collaboration with a local medical coordinator.

When patients are registered in a GACO, they are assigned a priority code. This code is determined by a nurse in collaboration with the local medical coordinator based on the urgency and complexity of the case. This priority system was set up so that patients whose health status requires urgent management can be enrolled with a family physician more rapidly. Standards regarding length of wait times for enrolment with a family physician according to priority codes are set by the MSSS and the FMOQ. Priority 1 indicates that the nurse has assessed the patient’s health status as requiring immediate medical management. Such would be the case, for example, of someone presenting complex pathologies or health status destabilization. For this patient category, the MSSS and FMOQ recommend referral to a family physician within 30 days. Patients identified as Priority 2 require medical management within the short term (less than three months), and those assessed as Priority 3 need to be seen within the medium term (less than six months). Priority 4 patients are considered non-urgent, but should be seen within a year. Priority 5 patients are in good health, with no known health problems; there is no recommended time frame for their referral to a family physician.

Patients are enrolled with a family physician through a process that respects medical manpower availability, the practice locations of physicians registered with GACOs, as well as the established priorities. The information regarding patients’ health status is documented and sent to the physicians. From the GACOs’ creation until November 2011, physicians received a payment of $103 for each patient designated as vulnerable who was enrolled through a GACO, but no payment for non-vulnerable patients. The amount was paid in two instalments: the first at the patient’s first medical visit, and the second when the patient was seen by the physician during the following year. Patients were considered vulnerable if they presented with one of 19 codes of vulnerability as defined by Quebec’s Health Insurance Board, the *Régie de l’Assurance-maladie du Québec* (RAMQ), based on the presence of certain medical diagnoses (e.g. diabetes, chronic obstructive pulmonary disease, mental health disorders).

### Change in financial incentives

In November 2011, the amount of these bonus payments was modified, and a new payment was introduced to further encourage family physicians to participate in GACOs and to increase the population’s access to family physicians. From then on, for enrolments processed through GACOs, family physicians received $100 for non-vulnerable patients and $208 for vulnerable patients. These payments are bonuses paid upon the patient’s first visit to the family physician. The government’s intent was to increase the number of patients enrolled with a family physician and to encourage enrolment of patients considered vulnerable. Family physicians’ participation in GACOs is voluntary.

### Study design: a before–after study of financial incentive changes

In this paper we report on the results of a before–after analysis of data from a provincial database of centralized waiting lists to determine whether changes could be observed in patients’ enrolment with family physicians after the financial incentives were modified. We analyzed the effect of that change (before–after) on the number of patients enrolled through centralized waiting lists. We analyzed data from a provincial administrative database used by professionals working in GACOs which tracks the number of patients registered and waiting as well as the number of patients enrolled with family physicians through GACOs. Within the database, we examined a five-year period (April 1, 2008, to March 31, 2013).

### Participants

#### Cohort study: all patients enrolled with a family physician through GACOs

The provincial database includes the 494,697 patients enrolled with family physicians through 87 GACOs across the province over the five-year period under study. This database excludes one region with six GACOS using another database. The study was approved by the Sherbrooke Hospital Research Ethics Board (ref. number CHUS14-091).

#### Variables and statistical analysis performed

We compared the number of patients enrolled with a family physician through GACOs before and after the changes in financial incentives were introduced in November 2011. The numbers of patients enrolled before and after were compared on three patient characteristics: presence of a vulnerability code, the source of referral for GACO registration, and the assigned priority code. The level of analysis was the number of patients compared for 65 periods of time corresponding to 25 working days each. Mixed regression models for repeated-measures were used to compare the numbers of patients enrolled with a family physician through GACOs before (time periods 1-47) and after the change (time periods 48-65), based on the three characteristics of interest. We used three general linear mixed models, which are parametric linear models appropriate for non-independent data, with SAS 9.3 software, regressing the number of enrolled patients on indicators of vulnerability, source of referral for GACO registration, and priority code. There were no control variables.

## Results

Table 1 presents the details of the three mixed regression models for repeated-measures for the patient characteristics analyzed (vulnerability, source of referral for GACO registration, and priority code). For each characteristic, the numbers of patients enrolled with a family physician before and after the change were compared using linear regression. The p-value represents the significance of the before–after difference for this group, with alpha = 0.05.

**Table 1:** **Numbers of patients enrolled with a family physician through GACOs before and after the changes in financial incentives by vulnerability, by source of registration in GACOs, and by priority code**

		**Before (Periods 1 to 47) N = 47**	**After (Periods 48 to 65) N = 18**	**p-value**
**All patients**				
All	Total	107 100	387 597	<.0001
	Average per period	1 139	10 767	
	CI	555 ; 1 724	8517 ; 10 738	
**Patients with vulnerability**				
Yes: patients had a least one vulnerability code*	Total	74 017	116 119	<.0001
	Average per period	1 575	6 451	
	CI	1 086 ; 2 064	5 661 ; 7 241	
No	Total	33 083	271 478	<.0001
	Average per period	704	15 082	
	CI	215 ; 1 193	14 292 ; 15 872	
**Source of referrals for GACO registration** (The main categories are presented)				
Hospitalization and ER	Total	3 283	3 449	0.58
	Average per period	70	192	
	CI	-159 ; 299	-178 ; 561	
Health professionals	Total	43 706	48 057	0.0002
	Average per period	131	343	
	CI	72 ; 190	248 ; 439	
Self-referral	Total	13 314	24 8039	<.0001
	Average per period	289	13 780	
	CI	128 ; 451	13 518 ; 14 041	
User	Total	33 466	71 359	<.0001
	Average per period	824	3 964	
	CI	662 ; 986	3 704 ; 4 226	
**Priority codes**				
Priority 1: less than 30 days	Total	11 224	11 032	<.0001
	Average per period	239	613	
	CI	10 ; 467	244 ; 982	
Priority 2: less than 3 months	Total	31 378	78 341	<.0001
	Average per period	668	4 352	
	CI	440 ; 896	3 983 ; 4 721	
Priority 3: less than six months	Total	40 303	89 243	<.0001
	Average per period	858	4 958	
	CI	629 ; 1 086	4 589 ; 5 327	
Priority 4: less than one year	Total	8 750	38 531	<.0001
	Average per period	186	2 141	
	CI	42 ; 415	1 772 ; 2 510	
Priority 5: no time frame recommended	Total	15 445	170 450	<.0001
	Average per period	329	9 469	
	CI	100 ; 557	9 100 ; 9 838	

*Patients were considered vulnerable if they presented with one of 19 codes of vulnerability as defined by Quebec's Health Insurance Board (RAMQ) based on the presence of certain medical diagnoses (e.g. diabetes, chronic obstructive pulmonary disease, mental health disorders).

### Change in volumes of patients enrolled with family physicians through GACOs

**Figure 1:**
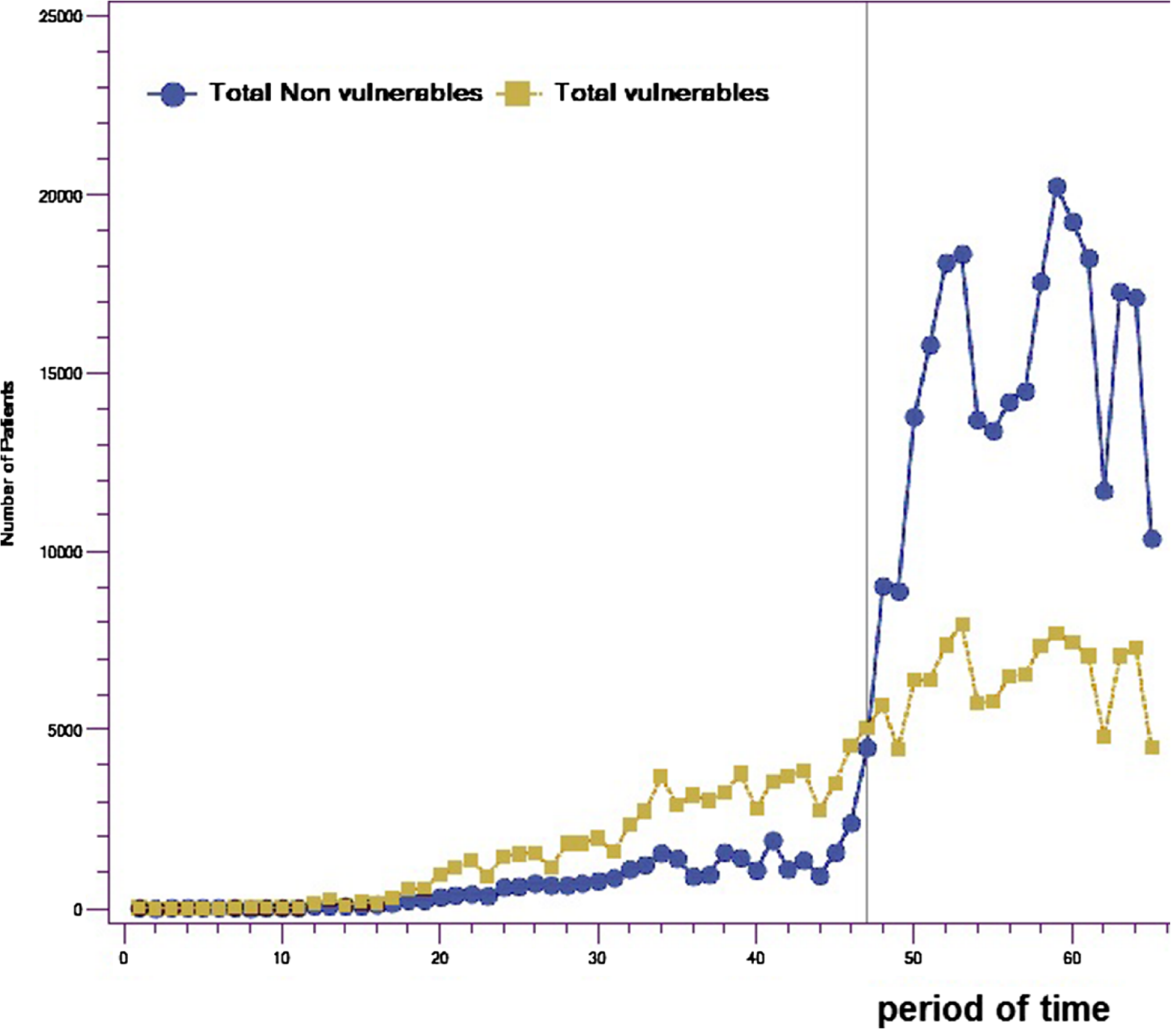
**Numbers of non-vulnerable patients and vulnerable patients enrolled with family physicians through GACOs.**

Figure 1 presents the number of patients enrolled with family physicians through GACOs over the five years under study, divided into 65 administrative periods of 25 working days each. The figure shows two phases: before and after the financial incentives were changed.

Over these five years, across the province of Quebec, 494,119 patients were enrolled with a family physician through GACOs. As seen in Figure 1, the number of patients enrolled increased substantially over time, especially after the introduction, in November 2011 (period 47), of the new financial incentive for enrolling patients considered non-vulnerable. This was when the government doubled the incentive for enrolling vulnerable patients (from $103 to $208) and added a $100 incentive for enrolling non-vulnerable patients. After the 47^th^ period, we observe a substantial increase in the number of non-vulnerable patient enrolments (p <0.001), whereas the number of vulnerable patients enrolled through GACOs increased only slightly (p <0.001). The temporary reductions observed in the total volume of patients enrolled through GACOs correspond to summer months and end-of-year holiday seasons. The increases in both vulnerable and non-vulnerable patients were significant, but the magnitude of change was much (21 times) larger for non-vulnerable patients. As presented in Table 1, before the change, the mean number of patients without a vulnerability code enrolled per period with family physicians was 703; this increased to a mean of 15,082 patients per period after the change.

Although, the absolute number of vulnerable patients enrolled slightly increased after the changes to financial incentives in November 2011, a significant decrease in the proportion of enrolled patients who were vulnerable was observed with a corresponding significant increase in the proportion of enrolled patients who were non-vulnerable. Before the new incentive to enrol non-vulnerable patients was introduced, nearly 70% of patients enrolled through GACOs were vulnerable patients; afterward, the proportion of enrolments of vulnerable patients dropped to 30%.

### Change in the source of referrals for GACO registrations

Referrals may be made to the GACO from different sources. Patients may put themselves on the list, either by telephoning, mailing in a form, or registering online. Patients may also be referred by a health professional following a clinic consultation, an ER visit, a hospitalization, or in another care context. When a referral for GACO registration comes from a family physician who is referring the patient to himself, this source is considered to be a self-referral by the family physician.

**Figure 2:**
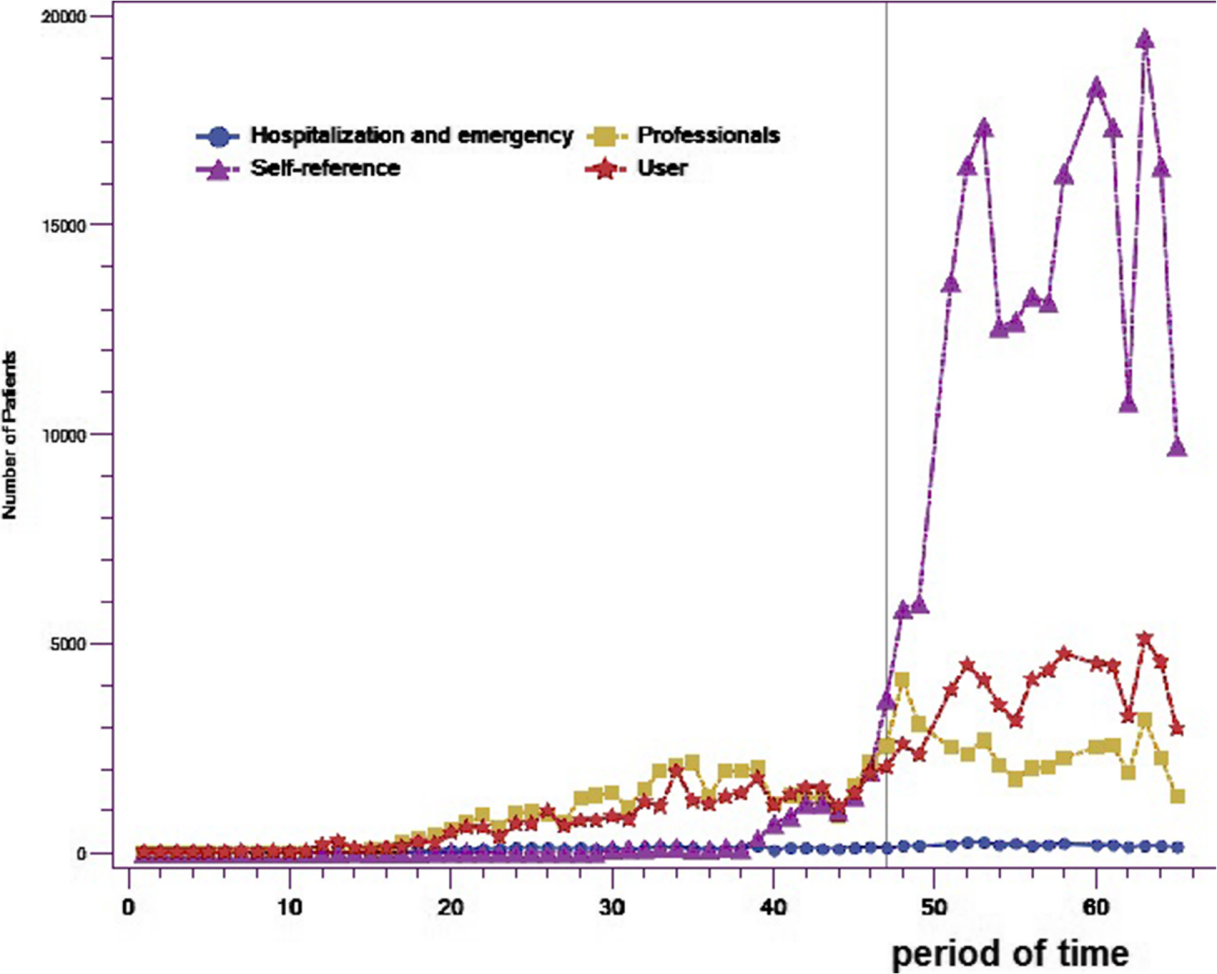
**Numbers of patients enrolled with family physicians through GACOs, by source of referrals for GACO registration.**

Figure 2 shows the increased use of physician self-referrals (dotted-line curve). Whereas less than 15% of patients came from physician self-referrals before the incentives were changed, that proportion rose to 70% on average. Table 1 shows that, before the change, a mean of 289 patients per period came from self-referrals, increasing to 13,780 afterward—an increase of 48 times. However, the number of patients coming from hospitalizations and emergency rooms was low before the change, with a mean of 69 patients per period, and did not increase significantly afterward (p = 0.58), rising only to a mean of 191 patients per period.

### Change in the priority codes of patients enrolled with family physicians through GACOs

As mentioned earlier, when patients are registered with a GACO, they are assigned a priority code (P1 to P5) based on a clinical assessment, with the highest priority being P1. It should be recalled that the amount of the financial incentive paid to physicians is based on whether or not the patient is designated as vulnerable; the incentive does not, however, take into account the assigned priority code.

**Figure 3:**
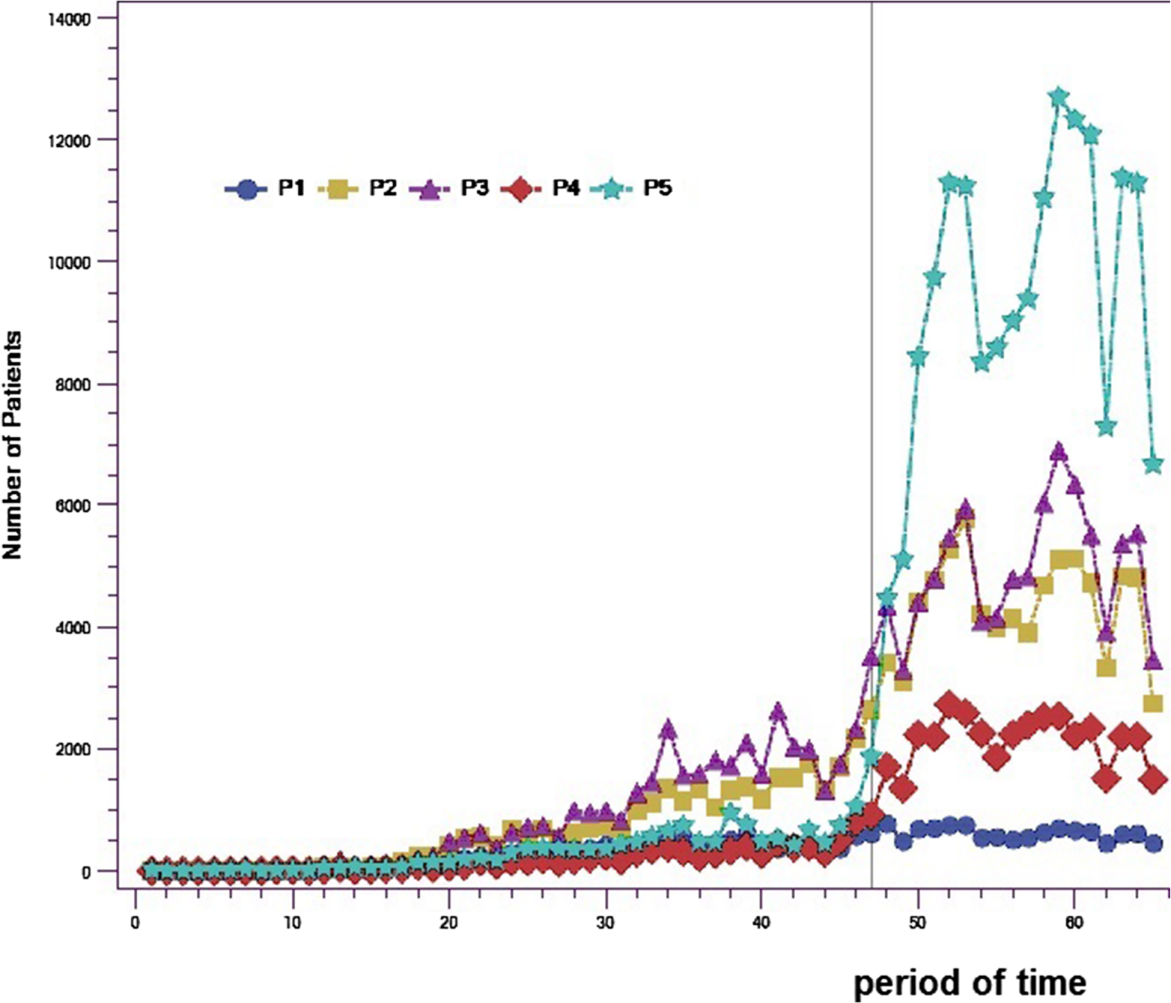
**Numbers of patients enrolled with family physicians through GACOs, by assigned priority codes.**

The results revealed a major change in the distribution of patients enrolled with a family physician through GACOs in terms of assigned priorities. The most striking change is the significant increase in the proportion of patients designated as priority 5, that is, patients considered to be in good health and not in need of immediate care. This clientele is illustrated in Figure 3 by the curve with stars. In effect, the results show that nearly 45% of the patients enrolled with family physicians through GACOs during the last year were patients assessed as being in good health, whose health status did not require even non-urgent care. Before the change, a mean of 328 patients enrolled per period were priority 5 compared to a mean of 9,469 patients per period after the change (p <0.001). Meanwhile, the number of patients designated as priority 1 changed only slightly over this time; these were the patients whose health status was considered to require prompt attention from a family physician. Before the change, a mean of 238 priority 1 patients were enrolled per period with a family physician through GACOs, while after it, that mean number increased only to 612 (p <0.09).

## Discussion

### Key results

Our results paint a mixed portrait of how well the GACOs’ objectives have been met through the use of financial incentives. These observations are important for structuring other experiences of centralized waiting lists.

### Interpretation

#### Did GACOs help increase the enrolment of real orphan patients?

GACOs were originally set up with the objective of increasing enrolments of orphan patients while giving priority of access to vulnerable patients. In the first phase of the GACOs’ existence, the volume of patients enrolled through this mechanism was low. GACOs were introduced in 2008, and it took at least two years before they were implemented all across the province, which explains the small number of patients enrolled with a family physician through that mechanism during that period. Our results showed a slight increase in the number of patients over the period prior to the change in financial incentives. However, as can be seen in the graphs presented, after the financial incentives were changed, the number of patients enrolled with family physicians through GACOs more than doubled in one year. In 2011–2012, 140,434 patients were enrolled with family physicians through GACOs. That number increased to 291,676 in 2012–2013, after the change in incentives. However, after November 2011, patients enrolled with family physicians through GACOs were mainly non-vulnerable and non-priority patients coming through physician self-referrals.

According to the MSSS’s database, in 2011–2012 and 2012–2013, 4,821,216 persons and 5,030,019 persons, respectively, were enrolled with a family physician, out of a total population of 8 million. Changes in the overall number of patients enrolled with family physicians in the population reflect the fact that every year new patients are enrolled, some patients die or relocate, and some family physicians retire. Between 2011–2012 and 2012–2013, the number of patients enrolled with a family physician in the population increased by 208,803, and over that period 291,676 patients were enrolled with a family physician through the GACO mechanism. This unexpected result suggests that the majority of the new patients enrolled with a family physician during this period came from GACOs.

Another phenomenon observed was the registration in GACOs of many patients of retiring family physicians. Prior to the existence of GACOs, when family physicians retired, their patients were informally transferred to other physicians. The GACOs thus created a more formal and costly transfer of patients within the healthcare system. For patients who are “orphaned” after the retirement of their family physician, GACOs might be helpful. However, mechanisms should be developed to help such patients, particularly the most vulnerable ones.

Usually, family physicians enrol new patients each year in the course of natural patient turnover due to death, relocation, or other life events. In a way, GACOs reward family physicians for a task they were already doing: taking on new patients. The idea behind GACOs was to help orphan patients, based on a priority assessment. This raises new questions: What proportion of patients enrolled through GACOs were actually patients who did not already have a family physician, that is, were real orphan patients? Having a family physician and being formally enrolled with a family physician are different. The GACO system encourages the enrolment of patients. However, patients may declare themselves as having a family physician without having signed any formal enrolment agreement. The GACO system may have provided an opportunity to formally enrol patients who were already being followed without formal enrolment. Does the GACO system remunerate physicians for seeing patients they would have seen anyway? This raises important efficiency issues. We are not able to link those patients with the data we used. However, it will eventually be possible to analyze whether patients enrolled through GACOs had already received medical services from that family physician prior becoming enrolled by analyzing the RAMQ’s medical care insurance billing data. This kind of analysis will be an important contribution to the analysis of the GACO policy’s impact.

#### What explains the non-enrolment of vulnerable patients?

Even when the payment to family physicians for taking vulnerable patients doubled and was more than twice the amount for non-vulnerable patients, family physicians showed a preference for enrolling non-vulnerable rather than vulnerable patients. This may reflect the fact that non-vulnerable patients are more prevalent in the pool of patients waiting in the GACOs. So, while the objective of increasing enrolments with family physicians was achieved by changing the incentive system, that of giving priority to vulnerable patients was not. The intention had been to enrol vulnerable patients first, ahead of patients with no known health problems (non-vulnerable patients) who do not need frequent attention. After the new financial incentives were introduced, there was a substantial increase in the enrolment of non-priority patients, but the volume of patients considered vulnerable and of higher priority did not increase at the same pace. This presented a paradox, in that the two objectives—increasing the number of patients and prioritizing vulnerable patients—appeared to be incompatible, or at least not achievable through the same means. Our results suggest that physicians tend to prefer receiving a lower amount per patient while enrolling more patients who are less demanding and probably require shorter medical consultations, over receiving a higher amount for vulnerable patients who require more care. This observation is in line with results from other studies [22,23]. As such, patients who are unwell will have a harder time finding family physicians, leading to problems of equity in access to care. According to several stakeholders and physicians we encountered, some clienteles, such as those with mental health problems and drug addictions, waited longer in GACOs before being matched with a family physician, and those delays increased after the new financial incentives were implemented. Also, newspapers have reported cases of discrimination against certain vulnerable clienteles such as the elderly and persons with mental health problems and drug addictions [24,25]. Is this because physicians want to minimize the time spent in consultations in a fee-for-service system? Or is it for other reasons, such as their own perceived lack of expertise for treating patients with complex health conditions?

#### What are the next policy options for optimizing the functioning of GACOs?

##### Enrolment of vulnerable patients only

In the current context, is it really advisable to set as an objective the enrolment of the entire population, when vulnerable patients have a greater need? As noted earlier, of Quebec residents without a family physician, nearly one-third said they did not need one. While the benefits of having a family physician are numerous for vulnerable clienteles [26-28], for others, such as those who are young and in good health, sustained contact with a physician has little measurable impact on their already good health status [29]. Currently, approximately 376,000 persons across the province are registered in GACOs and waiting to be enrolled with a family physician. That represents almost 11.87% of the 3,167,148 persons who are not enrolled with a family physician. Note that some patients do not want a family physician, others declare having a family physician but are not formally enrolled with them, and yet others are trying to find a family physician without registering in GACOs. About 25% of the patients registered in the GACOs and waiting to be enrolled with a family physician have been assessed by a health professional and identified as having at least one vulnerable condition. These patients are the ones who need to be followed regularly to ensure good management of their illness and close monitoring of their health status [30]. It would therefore seem appropriate to focus more on enrolling vulnerable populations.

##### Self-referral mechanism

The self-referral mechanism allows physicians to select the patients they will enrol through the GACOs instead of being linked with patients they have never seen. This practice of self-referral appears to short-circuit the objective for which the GACO system was created, which was to centralize patients’ requests on a shared list and to set access priorities based on the urgency and complexity of cases as assessed by health professionals. In Ontario, the neighbouring province, centralized waiting lists do not accept physician self-referrals [31]. In fact, in reaction to substantial cost increases related to GACOs, and the fact that almost 70% of new enrolments now come through self-referrals, in June 2013 the Quebec government put new rules in place. Physician self-referrals are now prohibited. Family physicians are limited to 150 patients enrolled through GACOs per year, and larger financial incentives ($250) are offered for enrolling “super vulnerable patients” (defined as having complex problems such as mental illness and substance use problems). It will be interesting to analyze the impact of these new rules and financial incentives on the numbers of patients enrolled through GACOs in the coming years.

### Generalizability

#### Incentives in the context of payments and care organization

Incentives should also be analyzed within their implementation context [32]. Financial incentives are often used for instrumental purposes in a rational world of personal interest [16]. Our results showed, however, that income maximization did not appear to be the only driver for physicians’ behaviours and suggested the need for a serious and thorough process of reflection to understand why physicians are reluctant to enrol vulnerable patients.

Furthermore, in Quebec, payments to physicians participating in GACOs occur in a context where fee-for-service remuneration is predominant and remuneration by capitated payment is relatively uncommon. Several studies have suggested that a mixed remuneration model combining fee-for-service and more generous capitated payments might be a more promising solution [22] for the management of complex clienteles.

Our study analyzed the case of GACOs in Quebec, but our results are quite illustrative of the reactivity of physicians’ practice to financial incentives. Using a longitudinal study design based on a provincial dataset covering a five-year period, our analysis enabled us to draw some important conclusions regarding physician behaviours. As such, we believe our results can be used to inform the design of centralized waiting lists in other healthcare contexts.

#### Limitations

The data were analyzed at the provincial level, and we did not have access to certain descriptive variables of the cohort as the sex, age, and patient location (urban vs. regional). All GACOs across the province were analyzed together, thereby limiting our capacity to analyze variations among GACOs. Some GACOs are being used to reassign the patients of retiring physicians. Some only take patients deemed vulnerable and identified as a high priority; others register all patients, regardless of health status. We were unable to perform multivariate regression analysis on these factors because our database did not collect such detailed information. This is a limitation of our study. However, the patient profiles are based on a standard definition of vulnerability (presence of at least one vulnerability code based on a list of 19 diagnoses). It will be interesting to do further analyses on various clienteles, such as patients with mental health problems, and to analyze differences in relation to numbers of vulnerability codes. That information is present in the local databases of GACOs but not aggregated at the provincial level. Also, because we analyzed the longitudinal database at the provincial level, variations in physician demand and capacity across different regions of the province could not be included in the analysis.

## Conclusion

This study showed that a change in financial incentives slightly increased the number of vulnerable patients being enrolled with family physicians and largely increased the number of non-vulnerable patients being enrolled with family physicians. The GACO experience demonstrates the need to monitor the effects of these incentives very closely and to react promptly to align them more closely with the objectives targeted. This experience also reveals the limitations of financial incentives in orienting medical practice. It would be important to anticipate the effects while considering the whole context of payments and care organization and to explore other medical practice organizational models that would foster more equitable management of the population’s health.
